# Creatine supplementation and muscle-brain axis: a new possible mechanism?

**DOI:** 10.3389/fnut.2025.1579204

**Published:** 2025-07-23

**Authors:** Felipe Ribeiro, Scott C. Forbes, Darren G. Candow, Pedro Perim, Fabio Santos Lira, Antonio Herbert Lancha, José C. Rosa Neto

**Affiliations:** ^1^Laboratory of Immunometabolism, Department of Cell Biology and Developmental, Institute of Biomedical Sciences, University of São Paulo, São Paulo, Brazil; ^2^Department of Physical Education Studies, Faculty of Education, Brandon University, Brandon, MB, Canada; ^3^Faculty of Kinesiology and Health Studies, University of Regina, Regina, SK, Canada; ^4^Faculty of Medicine, University of São Paulo, São Paulo, Brazil; ^5^Exercise and Immunometabolism Research Group, Post-graduation Program in Movement Sciences, Department of Physical Education, Universidade Estadual Paulista (UNESP), São Paulo, Brazil; ^6^Physical Education School, University of São Paulo, São Paulo, Brazil

**Keywords:** creatine, supplementation, myokines, BDNF, muscle-brain axis, skeletal muscle, brain

## Abstract

The brain and skeletal muscle have a high energy demand, of which creatine is an important regulator. Creatine acts as both a spatial and temporal energy buffer and reduces oxidative stress and inflammation. Creatine supplementation is well-recognized to enhance exercise performance, muscular strength and lean tissue mass, with emerging research showing benefits on cognitive function. Herein, we discuss the potential muscle-brain axis and the purported benefits of creatine supplementation on myokines, with a focus on brain-derived neurotrophic factor (BDNF). Myokines and the muscle-brain axis have been implicated in strength, endurance, neuroprotection, and cognitive performance, particularly in aging and clinical conditions. Creatine is a pleiotropic molecule and the mechanisms are multifactorial, however, they appear to be associated with improved bioenergetics, muscle hypertrophy, anti-inflammatory effects and on improved glucose metabolism. Despite the growing body of research on creatine, limitations such as variability in study designs, dosages, and individual responses need to be carefully interpreted. Further research is warranted to verify this hypothesis and to establish optimal supplementation protocols, particularly, in terms of its short-term and long-term implications for neuromuscular and cognitive performance.

## 1 Introduction

Creatine (methylguanidine acetic acid) is a nitrogenous compound synthesized endogenously from reactions involving the amino acids arginine, glycine and methionine in the liver and brain. Additionally, creatine can be obtained exogenously through the diet, from foods of animal origin, such as beef, fish or pork (63), or via a commercially available creatine supplement. Once endogenously synthesized or absorbed, creatine is transported into cells via a sodium-chloride dependent transporter, SLC6A8, against a concentration gradient. Inside the cell, about one-third of creatine remains free, while the remainder is phosphorylated to phosphorylcreatine (PCr). PCr donates its phosphate to ADP to resynthesize ATP through creatine kinase (CK) activity (1). This ATP-PCr system serves as a rapid energy buffer, especially during activities of high energy demand such as resistance exercise, by maintaining ATP levels in energy-demanding tissues like muscle, brain, and heart (2).

Beyond muscle bioenergetics, creatine appears to influence brain function. It is present in synaptic vesicles and may impact cortical neuron communication (3), while also stimulating mitochondrial activity in hippocampal neurons (4). Its potential neuroprotective effects are linked to oxidative stress modulation (5), antioxidant actions (6), electrophysiological changes (7), and neurodevelopment, are of growing interest. There is also evidence suggesting that creatine is a possible neurotransmitter in the Central Nervous System (CNS), due to its presence in synaptic vesicles, release upon stimulation, effects on cortical neurons and uptake into synaptosomes and synaptic vesicles (8).

In parallel, skeletal muscle is now recognized as an endocrine organ that releases myokines, that are cytokines and peptides that mediate crosstalk with distant organs including the brain (9, 10). As mentioned, creatine is an essential compound in cellular metabolism, especially in muscle cells, acting mainly in the rapid regeneration of ATP for muscle contractions, and myokines are released by the skeletal muscle following the muscle contraction. The communication between these molecules released by the skeletal muscle that can reach the brain can be named muscle-brain axis, which may offer a novel framework through which creatine exerts CNS effects. Thus, this mini-narrative review aims to explore the potential link between creatine supplementation and the muscle-brain axis mainly through myokine signaling.

## 2 Creatine supplementation: from sports to clinical applications

Creatine is widely recognized as one of the most effective and commonly used ergogenic aids to enhance exercise performance. In sport, creatine is recognized mainly for activities involving short bursts of high-intensity exercise or to improve resistance training adaptations (11). Mechanistically, creatine enhances exercise performance by increasing muscle PCr stores, which in turn facilitates rapid ATP resynthesis during high-intensity activities. This results in improved performance in activities such as sprinting and resistance training, where the ATP-PCr energy system predominates (1, 12, 13). Creatine supplementation combined with resistance training is associated with increased lean tissue or fat-free mass and muscular strength, likely due to enhanced training adaptations and recovery (13–15). Due to creatine’s capacity to improve anaerobic work performance during repeated high-intensity efforts, in theory, this could be advantageous for endurance sports like cross-country skiing, mountain biking, cycling, running and triathlon where changes in pace and surges may impact the race (16). Furthermore, creatine may benefit short-duration events where final bursts of effort are crucial, such as rowing, kayaking, and track cycling. Beyond creatine’s role in physical performance, research suggests that creatine may facilitate post-exercise recovery, support injury prevention and aid in thermoregulation (11).

Creatine supplementation also has potential benefits in various clinical contexts, including neurodegenerative disorders such as muscular dystrophy, Parkinson’s disease, Huntington’s disease and may contribute to neuroprotection in cases of concussions or spinal cord injuries (17). Other areas of interest include creatine’s use in managing diabetes, osteoarthritis, fibromyalgia, and age-related sarcopenia, as well as its protective effects against ischemic events in the brain and heart (18). Preliminary studies also suggest creatine’s potential in reducing the risk of depression and supporting maternal health during pregnancy (19, 20). Its safety profile is well-established, with no significant adverse effects reported in healthy individuals, although caution is advised in those with pre-existing kidney conditions (21). Overall, creatine supplementation is a versatile and pleiotropic dietary strategy that may benefit both sport performance and improve health in clinical settings.

## 3 Muscle as an endocrine organ: the muscle-brain axis

Skeletal muscle functions as an endocrine organ by releasing myokines, which are signaling proteins produced and secreted by myocytes in response to muscle contractions (10). These myokines facilitate communication between muscle and other organs, including the brain, forming what is referred to as the muscle-brain axis (22). Among the most studied myokines in this context are brain-derived neurotrophic factor (BDNF), cathepsin B, interleukin-6 (IL-6), insulin-like growth factor-1 (IGF-1), irisin and lactate. These molecules can cross the blood–brain barrier and have been shown to promote neuronal proliferation, synaptic plasticity, and cognitive improvements, as well as ameliorate behavioral alterations (22–24).

BDNF support differentiation, maturation, and survival of neurons in the CNS, so it plays a central role in synaptic plasticity, neurogenesis, and cognitive enhancement (25). Cathepsin B stimulates BDNF expression and contributes to improved memory and hippocampal function (24). IL-6, whose levels rise significantly during exercise, exhibits pleiotropic properties, acting either pro- or anti-inflammatory depending on the milieu, and is implicated in mood regulation and energy balance (23). IGF-1, transported from the periphery to the brain, has been shown to enhance neurogenesis and protect against neuronal apoptosis (26). Irisin, which is a molecule cleaved from fibronectin type III domain containing 5 (FNDC5), a precursor protein expressed in muscle, is also expressed in the hippocampus, where it stimulates the expression of the BDNF in this area that is associated with learning and memory (27). Lactate, traditionally viewed as a metabolic byproduct, is now recognized as a signaling molecule that modulates brain-derived neurotrophic pathways associated to neural development, neuroplasticity, blood–brain barrier maintenance, and lysosomal acidification (28).

**Figure 1:**
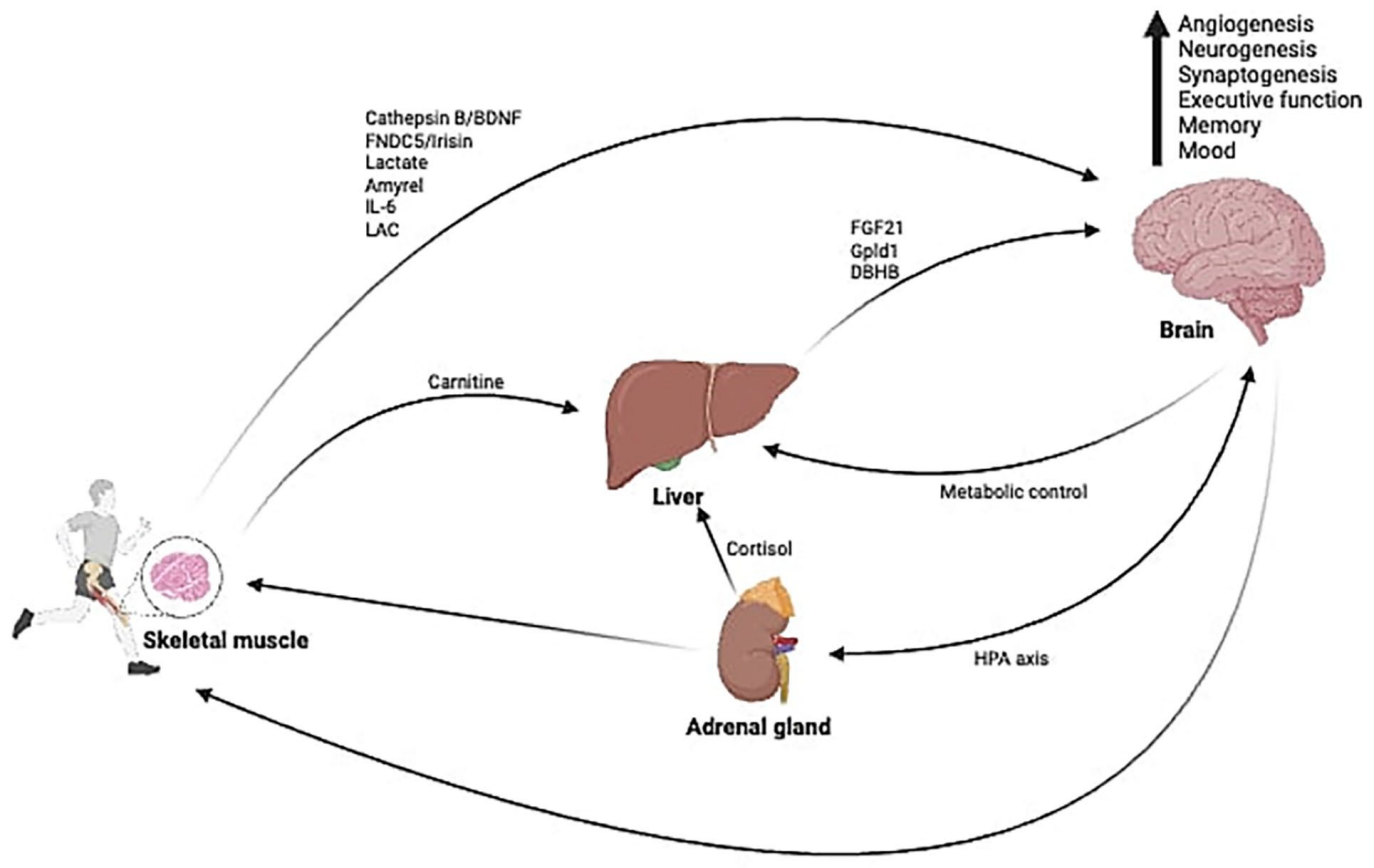
Illustration about the ability of muscle contraction during exercise to secrete myokines with the capacity to communicate directly with the brain or being mediated by the liver and promoting several cognitive benefits.

Overall, these myokines demonstrate how skeletal muscle, through contraction-induced secretion, can directly affect brain physiology and cognitive health (22, 24, 29). This area of research continues to evolve, with ongoing studies aimed at elucidating the precise mechanisms and therapeutic potential of muscle-derived signals (see Figure 1).

## 4 Creatine and myokines: a new possible mechanism?

Myokines mediate communication between muscles and other organs, such as the brain, adipose tissue, liver, bone, pancreas, intestine and skin, in addition to having effects on the muscle itself. In this way, myokines exert their effects on cognition, lipid and glucose metabolism, browning of white adipose tissue, bone formation, endothelial cell function, hypertrophy, skin structure and tumor growth (30). Creatine is an essential compound in cellular metabolism, especially in muscle cells, acting mainly in the rapid regeneration of ATP for muscle contractions. A possible relationship between creatine and myokine production occurs through different means, such as the mechanisms outlined below.

### 4.1 Increased muscle energy

Creatine supplementation increases the availability of ATP during intense or demanding exercise (Harris et al., 1992). When contraction stimulus is received by muscle cells, ATP and Ca2+ are demanded for muscle function. The available ATP lasts only for a few contractions, so the cell uses creatine phosphate to resynthesize ATP (31). One of the manners to provide more creatine, so this pathway can maintain is through creatine supplementation and this increase in the availability of ATP can possibly enhance muscle energy for contraction and modify the production of myokines, such as IL-6 (interleukin-6), which are signaling proteins involved in cellular communication and the muscle recovery process (11).

IL-6 is a myokine produced that helps regulate cellular metabolism, increasing when there is a reduction in ATP synthesis. Thus, creatine supplementation could, in theory, reduce IL-6 levels, which in turn has such effects on the CNS, such as in major depressive disorder prognosis and therapeutic responses, and may affect a wide range of depressive symptomatology (32). Moreover, short-term creatine supplementation is able to induce the increase in strength, endurance, mean power output in repeated bouts from sprint tests and this short-term protocol was able to increase the mean power and speed, mainly in the second half of sprints bouts in treadmill (33–35, 61). Interestingly, all of these studies did not show a difference in lactate concentration or other myokines into blood. It is significant because the lactate is an important metabolite associated to muscle energy and a myokine that induces directly the increase in the BDNF (36). We hypothetize that greater ATP availability as a consequence of creatine supplementation increases myokine production, which should be evaluated by future studies.

### 4.2 Muscle hypertrophy and inflammatory response

Creatine is established to augment resistance training gains in lean tissue mass. Mechanistically, creatine promotes an anabolic environment in muscle cells, which may lead to increased translation of muscle proteins. The magnitude of the myokine-release response after muscular contraction varies with the intensity, type, and volume of exercise performed (37). Myokines, such as IL-6, are produced in response to exercise and play crucial roles in muscle recovery and growth (38). Furthermore, creatine may affect various signaling pathways that control myokine production, including the mTOR pathway, which plays a key role in muscle growth and protein synthesis (39). By modulating the secretion of myokines such as myostatin and IGF-1, creatine may indirectly influence brain-related processes including neurogenesis, synaptic plasticity, and cognitive function (29, 62). In particular, higher intramuscular IGF-1 levels, as observed following creatine supplementation (40), have been associated with enhanced neurogenesis and synaptic plasticity, owing to the ability of IGF-1 to cross the blood–brain barrier. Additionally, creatine can alter the expression of myogenic regulatory factors, enhancing satellite cell activity and muscle regeneration (41), which may contribute to the muscle-brain cross-talk via exercise-induced myokine release. These potential pathways suggest that creatine may play a role not only in muscle anabolism, but also in modulating systemic signals that reach and influence the brain.

From an anti-inflammatory and anti-catabolic perspective, creatine can influence the inflammatory response by modulating the production of pro-inflammatory and anti-inflammatory myokines. This modulation can impact muscle recovery and adaptation to exercise, promoting a balance between inflammation and recovery (42). This modification in the inflammatory response, with reduction of muscular inflammation after exhaustive exercise, is capable of reducing plasma inflammatory markers, such as prostaglandin E2 (PGE2), which may act on the CNS and affect the muscle-brain axis (43).

### 4.3 Effects on insulin metabolism

**Figure 2:**
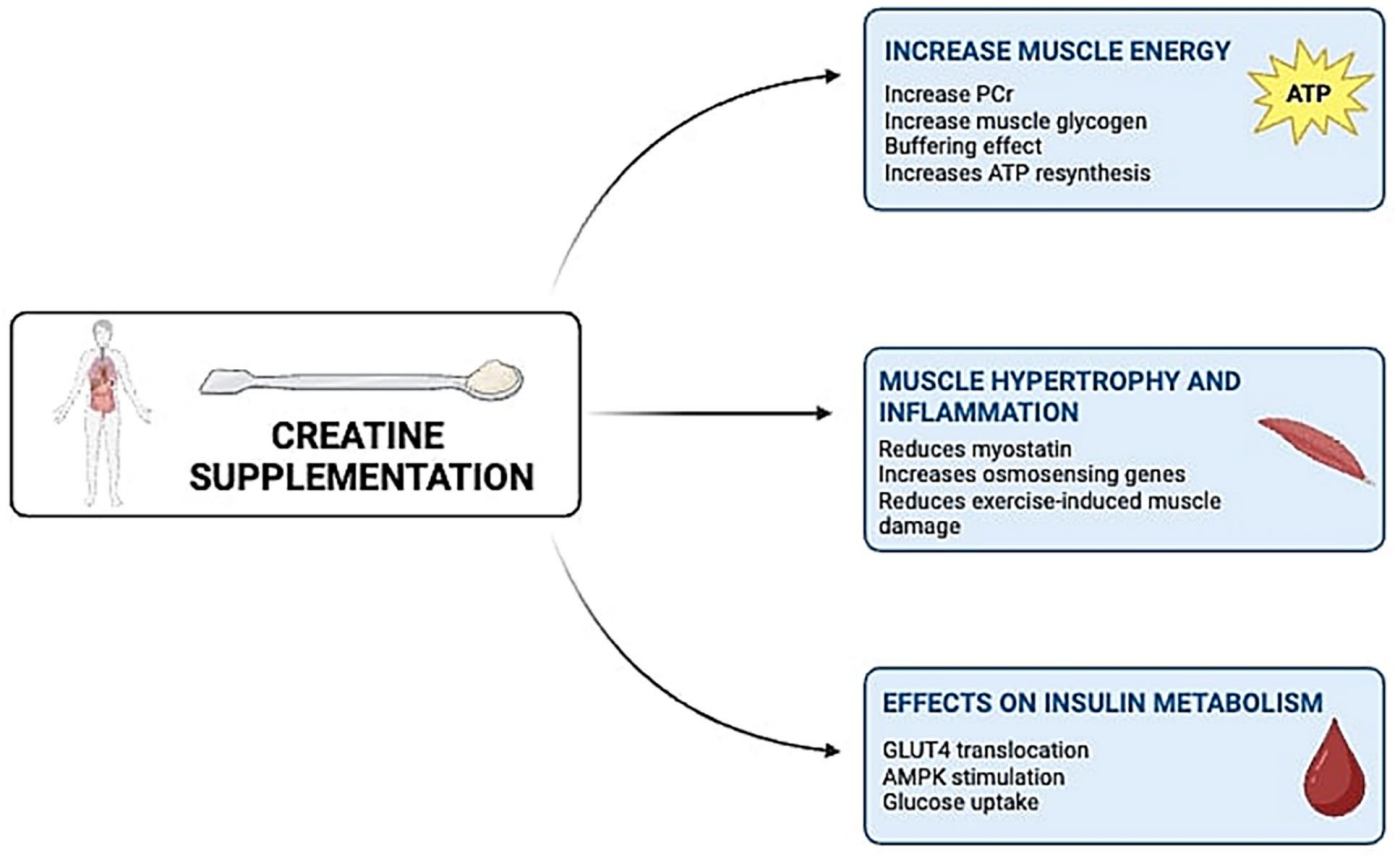
Multiple mechanisms that creatine supplementation in humans can promote metabolic responses. PCr, phosphocreatine; ATP, adenosine triphosphate; AMPK, adenosine monophosphate-activated protein kinase.

Creatine supplementation has been associated with improved insulin sensitivity (60), which may modulate the secretion of key myokines involved in muscle-brain communication. Improved insulin signaling mediated by creatine supplementation enhances glucose uptake via GLUT-4 translocation in skeletal muscle (44), which we hypothetize that can potentiate the exercise-induced release of myokines such as irisin, IL-6 and BDNF. Irisin is upregulated by exercise and insulin signaling, and crosses the blood–brain barrier to stimulate BDNF expression in the hippocampus, promoting synaptic plasticity, memory, and neurogenesis (45, 46). IL-6, whose secretion is also influenced by insulin sensitivity and muscular glucose uptake, plays a dual role in modulating neuroinflammation and energy metabolism in the brain (23, 47). Thus, the creatine-induced enhancement of insulin sensitivity could contribute to a favorable myokine profile, indirectly supporting brain health by amplifying neuroprotective and neuromodulatory signaling pathways associated with the muscle-brain axis (see Figure 2).

## 5 BDNF and creatine supplementation

Cytokines have several physiological effects in humans, including IL-6, interleukin-10 (IL-10), tumor necrosis factor-alpha (TNF-alpha), adiponectins and BDNF (10). The secretion of these cytokines is influenced by physical exercise, as exercise stimulates the release of neurotransmitters and neurotrophins (such as serotonin and dopamine) in an activity-dependent manner, which acutely enhances neural function and induces a cascade of events that promote structural and functional plasticity of the brain (48). Thus, creatine supplementation can influence key neurotransmitter systems, such as serotonin and dopamine, which are fundamental to mood regulation (49). These mechanisms suggest that creatine might help reduce depressive symptoms by restoring energy balance in brain cells and protecting them from neuroinflammation and oxidative damage. This set of mechanisms contributes to brain plasticity, with BDNF being one of the main mechanisms responsible for increasing neurogenesis and memory (50). BDNF is a protein that belongs to the neurotrophin family and is abundantly expressed in the hippocampus and cerebral cortex (51). BDNF plays a role in the formation and maintenance of neural synapses, as well as the ability of the CNS to adapt and regenerate to potential damage (52).

Approximately 70–80% of the BDNF present in the blood is produced by the brain at rest and during physical exercise (51). Therefore, increasing BDNF secretion is extremely important, and one of the main stimulants is exercise or muscular contractions. Regarding its relationship with physical exercise, acute exercise has been able to promote a temporary improvement in cognitive function, while long-term exercise training is associated with the stimulation of brain plasticity, improvement of brain function and prevention of neurological disorders (53). The most robust evidence available indicates that acute aerobic exercise promotes a transient increase in circulating levels of BDNF. Recently, Edman et al. (36) showed that the acute exercise increases the expression of pro-BDNF and the infusion of lactate induces the huge elevation in mature form of BDNF. In contrast, chronic exercise training appears to have variable effects on basal concentrations of BDNF (53, 54).

In the CNS, BDNF is primarily synthesized in the hippocampus, but can also be found in the cerebral cortex, midbrain, thalamus, hypothalamus, pons, and medulla oblongata (55). Thus, due to its low molecular weight (13 kDa) in its mature form, BDNF can cross the blood–brain barrier, and variations in the concentration of circulating BDNF likely originate from neurons and glial cells of the CNS (51). As such, BDNF plays an important role in neuronal survival and growth, acts as a neurotransmitter modulator and participates in neuronal plasticity, essential for learning and memory (56).

As previously mentioned, creatine can impact muscle function, which appears to be associated with improved training volume via increasing energy capacity and calcium uptake in the sarcoplasmic reticulum, or through the main pathways related to protein kinetics and protein degradation (57). The link between creatine and BDNF release may be explained through multiple interconnected pathways. First, creatine supplementation enhances phosphocreatine availability and ATP resynthesis, supporting higher training volumes and intensities, which are known stimuli for myokine release, including BDNF.

One specific mechanism involves the upregulation of Peroxisome Proliferator-Activated Receptor Gamma Coactivator 1 Alpha (PGC-1α) in skeletal muscle during exercise, a key regulator of mitochondrial biogenesis and oxidative metabolism, which also stimulates the expression of FNDC5, the precursor of irisin (46). Irisin, in turn, crosses the blood–brain barrier and induces BDNF expression in the hippocampus, contributing to neuroplasticity and cognitive improvements (45). Moreover, creatine may influence calcium signaling and mTOR pathway activation within muscle fibers, both of which are involved in activity-dependent myokine expression and may contribute to the enhanced release of neurotrophic factors like BDNF (64, 65). Thus, creatine’s ergogenic effects can indirectly support neuromodulation through myokine-mediated signaling from muscle to brain.

Pazini et al. (58) evaluated the antidepressant effect of creatine in mice with corticosterone-induced depression (20 mg/kg, orally, for 21 days). After treatment, creatine (10 mg/kg) and ketamine (1 mg/kg) reduced the immobility time in the tail suspension test, while fluoxetine (10 mg/kg) was not effective. The inhibitors of the PI3K/Akt/mTOR pathway (wortmannin and rapamycin) abrogated the antidepressant effects of creatine and ketamine. Both compounds restored BDNF levels in the hippocampus and increased synaptic proteins, such as PSD95. These results suggest that creatine, like ketamine, exerts a rapid antidepressant effect through the PI3K/Akt/mTOR pathway and BDNF modulation, being a potential alternative for the treatment of depression in mice.

More recently, Sherpa et al. (59) analyzed the efficacy and safety profile of oral creatine monohydrate combined with cognitive-behavioral therapy (CBT) on depression. An 8-week pilot, double-blind, randomized, placebo-controlled protocol was conducted in 100 adults with depression. Patient Health Questionnaire-9 (PHQ-9) scores improved in both groups over 8 weeks, with a significantly greater reduction in the creatine+CBT group. Findings suggest that 5 g of creatine supplementation for 8 weeks is a safe and potentially effective adjunct to CBT for depression, warranting further investigation in larger trials. However, there are still no clinical trials that have evaluated whether creatine supplementation influences the production of myokines, such as BDNF.

## 6 Future directions and conclusion

The relationship between creatine supplementation, physical exercise and the concentration of myokines, such as IL-6, BDNF, among others, is a topic of increasing relevance. Beyond the muscle, creatine can influence the health of the CNS, which may be associated with the modulation of the levels of BDNF, IL-6, IL-10, serotonin and dopamine. IL-6 and IL-10 have crucial roles in the inflammatory response and muscle recovery.

As future directions, it would be important to investigate if creatine supplementation, by increasing energy availability in cells, may favor the production of IL-10 and attenuate the negative effects of IL-6 in situations of oxidative stress and muscle inflammation. Furthermore, the relationship between creatine and neurotransmitters such as serotonin and dopamine warrants attention. So investigating the effects of creatine on cytokines and neurotransmitters is relevant to understanding creatine’s implications for health and physical performance, in addition to enhancing nutritional intervention and physical training strategies. Also, no clinical trials evaluated whether creatine supplementation has the potential to amplify BNDF effects, acting synergistically with physical exercise. The study of the interaction between creatine supplementation, exercise and BDNF levels can provide valuable outcomes for health promotion and prevention of diseases that affect the CNS. Finally, this interaction could be investigated in different interventions (e.g., dosage, time of administration, and population) because it can be relevant mainly as an adjuvant treatment of depression, sarcopenia, mood and neurological disorders, such as Alzheimer disease. There is a gap in the scientific literature that requires an investigation of the hypothesis that creatine supplementation can modify the expression of myokines released by muscle contraction induced by physical exercise.

In conclusion, we propose that creatine possible effects on the muscle-brain axis are a new possible mechanism, potentially modifying the production of BDNF and other neurotransmitters. The relevance of this field lies in the possibility of developing intervention strategies that can benefit mental and physical health, contributing to a better understanding of the relationship between nutrition, physical exercise and neurological function.
